# MiR-29c regulates the expression of miR-34c and miR-449a by targeting DNA methyltransferase 3a and 3b in nasopharyngeal carcinoma

**DOI:** 10.1186/s12885-016-2253-x

**Published:** 2016-03-15

**Authors:** Man Niu, Dan Gao, Qiuyuan Wen, Pingpin Wei, Suming Pan, Cijun Shuai, Huiling Ma, Juanjuan Xiang, Zheng Li, Songqing Fan, Guiyuan Li, Shuping Peng

**Affiliations:** Hunan Provincial Tumor Hospital and the Affiliated Tumor Hospital of Xiangya School of Medicine, Central South University, Changsha, 410013 China; Cancer Research Institute, School of Basic Medical Science, Central South University, Changsha, 410078 China; Department of Pathology, Second Xiangya Hospital, Central South University, Changsha, 410011 China; Guandong Provincial Yuebei People’s Hospital, Shaoguan, 512025 China; Orthopedic Biomedical Materials Institute, Central South University, Changsha, 410083 China

**Keywords:** Nasopharyngeal carcinoma, miR-29c, miR-34c, miR-449a, DNA methyltransferase

## Abstract

**Background:**

Nasopharyngeal carcinoma (NPC) is prevalent in South East Asia and Southern China particularly, despite the reported 5-year survival ratio is relative higher than other deadly cancers such as liver, renal, pancreas cancer, the lethality is characterized by high metastatic potential in the early stage and high recurrence rate after radiation treatment. MicroRNA-29c was found to be down-regulated in the serum as well as in the tissue of nasopharyngeal carcinoma tissue.

**Methods:**

In this study, we found accidentally that the transfection of pre-miR-29c or miR-29c mimics significantly increases the expression level of miR-34c and miR-449a but doesn’t affect that of miR-222 using real-time quantitative PCR in nasopharyngeal carcinoma cell lines. To explore the molecular mechanism of the regulatory role, the cells are treated with 5-Aza-2-deoxycytidine (5-Aza-CdR) treatment and the level of miR-34c and miR-449a but not miR-222 accumulated by the treatment. DNA methyltransferase 3a, 3b were down-regulated by the 5-Aza-CdR treatment with western blot and real-time quantitative PCR.

**Results:**

We found that pre-miR-29c or miR-29c mimics significantly increases the expression level of miR-34c and miR-449a. We further found DNA methyltransferase 3a and 3b are the target gene of miR-29c. Restoration of miR-29c in NPC cells down-regulated DNA methyltransferase 3a, 3b, but not DNA methyltransferase T1.

**Conclusions:**

The regulation of miR-29c/DNMTs/miR-34c\449a is an important molecular axis of NPC development and targeting DNMTs or restoring of miR-29c might be a promising therapy strategy for the prevention of NPC.

## Background

Nasopharyngeal carcinoma (NPC) is prevalent in South East Asia and Southern China particularly. Despite the reported 5-year survival ratio is relative higher than other deadly cancer such as liver, renal, pancreas cancer, the lethality is characterized by high metastasis in the early stage and high recurrence rate after radiation treatment. Due to the secluded anatomical sites, early symptom of patients is not typical, 80 - 90 % patients with NPC are diagnosed until the late advanced stage. EB virus infection, genetic factors, environmental and diet factor are widely recognized to be associated with the etiology of NPC carcinogenesis [1, 2]. However, recent studies have found that genome-wide epigenetic modifications in tumor associated gene are also involved in this process [3–5].

Epigenetic modification refers to the changes in gene expression, but not a genetic change in the DNA sequence, and can be stably transmitted through meiosis in the process of growth and cell proliferation. Epigenetic factor has been proved to play an important role in the carcinogenesis and development of nasopharyngeal carcinoma (NPC). Detection of epigenetic modifications can serve as molecular context of NPC and it is advantageous in the prognosis of NPC. The regulation of the epigenetic modification is reversible so that different intervention measures in epigenetic aspect may be used as a novel strategy to treat NPC, as well as the development of novel NPC radiotherapy sensitizing agent and novel drugs.

MicroRNAs (miRNAs), small non-coding RNA, exist in many organisms and play a important role in the regulation of protein expression by binding the 3′-untranslated region (3-UTR) of their target mRNAs through completely or incompletely complementary seed sequences and assembled in RNA-induced silencing complex(RISC), mediating the degradation of mRNA or the blockade of the translation of encoded protein. Abnormal expression of miRNAs has been demonstrated in most tumor types including NPC [6–8]. In previous studies of our laboratory as well as other research groups, miR-29c was found to be down-regulated in the serum of NPC patients [9–12], while, the effect of miR-29c and the pathways in which miR-29c works during the development and progression of NPC are not well defined. Therefore, in this study, we investigated the biological functions and molecular mechanism of miR-29c in NPC, which may help to further elucidate the roles of miRNAs in the development of NPC and provide a novel candidate target for therapeutic strategies for NPC.

In this study, we accidently found that pre-miR-29c transfection in nasopharyngeal carcinoma increased the expression of miR-34c and miR-449a. In order to seek for the molecular mechanism of this event, we hypothesized that miR-29c down-regulated DNA methytranferases (DNMTs), which catalyze the addition of a methyl group to the cytosine residue of CpG nucleotides. In NPC tissue, the down-regulation of miR-29c leads to the high level of DNMTs, which further promote the methylation of the CpG islands of tumor suppressors such as miR-34c and miR-449a. Our experimental data showed that epigenetic modifications of miR-34c and miR-449a are affected by the DNMTs, especially DNMT3a and DNMT3b.

## Methods

### Cells and cell culture

Human nasopharyngeal carcinoma cell lines, HNE-1,CNE-2,C666-1 and the immortalized human nasopharyngeal epithelial cell, NP69 were described previously [13, 14]. The NPC cell lines were maintained in 1640 (Gibco, Grand Island, NY, USA), supplemented with 10 % fetal bovine serum (FBS) (Gibco, Grand Island, NY,USA) and 1 % penicillin-streptomycin-glutamine (Gibco, Grand Island, NY,USA) at 37 °C and 5 % CO_2._ The nasopharyngeal epithelial cell line NP69, which is immortalized with an SV40 T-antigen, was a kind gift from Professor Sai Wah Tsao of the Department of Anatomy, University of Hong Kong, China, and was maintained in keratinocyte-serum free medium (Invitrogen, Carlsbad, CA, USA) with the addition of growth factor supplements (Life Technologies, Gaithersburg, MD, USA) [15].

### Drug treatment

Cells were incubated with the 5-Aza-2′-deoxycytidine(5-Aza-CdR) (10 μM) (Sigma, MO, USA) for 96 h, with or without Trichostatin A(TSA) (10 μM) (Sigma, MO, USA) or TSA alone for the last 24 h.5-Aza-CdR is methylation methytranferase inhibitor, an epigenetic modifier that inhibits DNA methyltransferase activity which results in DNA demethylation (hypomethylation) and gene activation by remodeling “opening” chromatin. Genes are synergistically reactivated when the demethylation is combined with histone hyperacetylation. Trichostatin A is a histone deacetylase inhibitor.

### Pre-miRNA constructs and miRNA mimics transfection

Pre-miR-29c or scramble cDNA together with restriction enzyme sites were inserted into pSuper vector (OligoEngine,WA,USA) and transformed into *Ecoli* JM109. The clones with positive inserts were subjected to the plasmids extraction and confirmed to be correct by DNA sequencing. Cells were seeded in 6-well dish (4*10^6^cells/well) the day before and were transfected with scramble pSuper or pre-miR-29c/pSuper with Lipofectamine™ 2000 (Invitrogen, Carlsbad, USA) according to the manufacturer’s instructions. Forty-eight hours after the transfection, the expression of miR-29c, miR-34b, miR-449a was detected by real-time PCR, and the expression of DNMT3a, 3b, T1 was tested by real-time PCR and Western blotting.

### Quantitative real time PCR (qRT-PCR)

Total RNA was extracted using miRNeasy Mini kit (Qiagen, Germany) according to the manufacturer’s instructions. For miRNA expression analysis, cDNA was synthesized using miScript II RT Kit (Qiagen, Germany). A PCR analysis was performed using miScript SYBR Green PCR Kit (Qiagen, Germany). Hsa-miR-29c-1 miScript Primer, Hsa-miR-34c-1 miScript Primer, Hsa-miR-222-1, Hsa-miR-449a-1 miScript Primer (Qiagen, Germany) were used and RNU6 (Qiagen, Germany) acted as an internal control. The PCR cycle parameters were as follows: 95 °C for 15 min, 39 cycles of denaturation at 95 °C for 15 s, annealing at 50 °C for 30s, and extension at 70 °C for 30s. For mRNA expression analysis, cDNA was synthesized using cDNA reverse transcription kit (Thermo Fisher Scientific, MA, USA) and a PCR analysis was performed using QuantiFast SYBR Green PCR Kit following the manufacturer’s instructions. The PCR cycle parameters were as follows: denaturation at 95 °C for 5 min, 39 cycles of denaturation at 95 °C for 10s, annealing at 60 °C for 30s, and extension at 72 °C for 30s. DNMT3a, 5′ primer (5′-CCGGA ACATT GAGA CATCT-3′) and 3′ Primer (5′-CAGCAGATGGTGCAGTAGGA-3′); DNMT3b, 5′ primer (5′-GGAGA CTCAT TGGAG GACCA; and 3′ Primer (CTCGG CTCTG ATCTT CATCC-3′); DNMT1, 5′ primer (5′-GAGCCACAGATGCTGACAAA-3′) and 3′ primer (5′-TGCCA TTAACACCACCTTCA-3′). β-actin, 5′ primer(5′-CCTATCGAGCATGGAGTGGT-3′) and 3′ Primer (5′-CTGAGGCATAGAGGGACAGC -3′), β-actin acted as internal control. These experiments were performed according to the manufacturer’s protocol of Bio-Rad CFX96 System.

### Western blot analysis

Cells were harvested at the indicated time and rinsed tweic with cold PBS. Cell extracts were prepared with lysis buffer containing 50 mM Tris–HCl, pH7.5, 150 mM NaCl, 2 mM EDTA, 1%Triton, 1 mM phenylmethylsulfonyl fluoride, and protease inhibitor mixture(Roche, USA) for 20 min on ice. Lysates were cleared by centrifugation at 14,000 rpm at 4 °C for 10 min. Supernatants were collected, and protein concentrations were determined by Pierce BCA Protein Assay (Pierce, USA). The proteins samples were separated by sodium dodecyl sulfate–polyacrylamide gel electrophoresis (SDS-PAGE) in 10 % (wt/vol) polyacrylamide gels and transferred to nitrocellulose membrane (Millipore, USA). After blocking with 5 % non-fat dry milk for 1 h at room temperature, the membrane was incubated with the primary antibodies in 5 % non-fat dry milk overnight at 4 - 8 °C. The following antibodies were utilized: anti-DNMT3a mouse polyclonalantibody (Santa Cruz, USA), anti-DNMT3b rabbit polyclonal antibody (Anbo, USA), anti-DNMT1 rabbit polyclonal antibody (Santa Cruz, USA), anti-β-actin mouse polyclonal antibody (Abclonal, USA). Membranes were washed and incubated with horseradish peroxidase-conjugated secondary anti-mouse antibody or anti-rabbit antibody (CST, USA). After additional washes with phosphate-buffered saline, the band signals were visualized and quantified with chemiluminescence kit (AidLab, China).

### Immunohistochemical staining and evaluation

The paraffin sections of NPC tissue microarray were collected from the patients of the Pathology Department of the Second Xiangya Hospital of Central South University between 2007 - 2014. The tissue slides were heated 65 °C for 1 h, and deparaffinized in xylene and rehydrated through graded alcohols (100, 90, 70 and 50 % alcohol; 5 min for each). For antigen retrieval, tissue slides were incubated in sodium citrate buffer (0.01 M, pH 6.0) for 20 min in a household Pressure cooker. After cooling to room temperature, the slides were washed in PBS (150 mM sodium chloride, 150 mM sodium phosphate, pH 7.2). The endogenous peroxidase activity was removed by incubating with 3 % hydrogen peroxide for 10 min and was blocked in normal goat serum (Maixin, China) for 30 min. The primary antibodies (anti-DNMT1, anti-DNMT3a and anti-DNMT3b) were applied at 4 °C overnight. Polymerized HRP and anti-rabbit IgG (Maixin, China) were added according to the manufacturer’s instructions. A color reaction was developed using DAB Color Developing Reagent Kit (Boster, China), and all of the slides were counterstained with hematoxylin staining kit. Negative control slides were included in the experiment. The immune histochemical staining of these sections was scored microscopically (Olympus, Japan) at × 400 magnification in all of the available tumor cells or epithelial cells meeting the typical morphological criteria by 3 pathologists using the qualitative scale that is described in the literature. The number of cells staining was scored as 0 (no staining), 1 + (<1/3 positive cells), 2 + (>1/3 and < 2/3 positive cells) and 3 + (>2/3 positive cells). The intensity of staining was scored from1 + (weak) to 3 + (strong). The immune reactive score was categorized into three groups by comprehensive evaluation of the percentage of positive cells and staining intensity.

## Results

### Hsa-miR-29c is down-regulated in NPC cell lines and NPC tissues, correlated with clinical stage of NPC

**Fig. 1:**
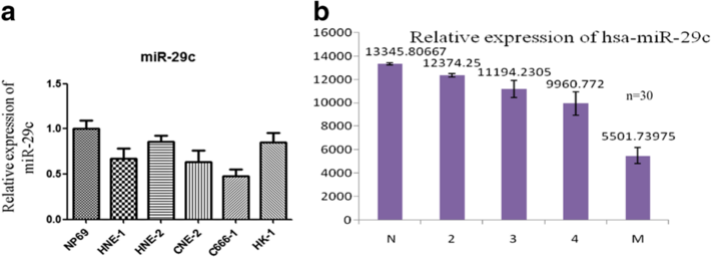
The expression of miR-29c is down-regulated in nasopharyngeal carcinoma cell lines and tissues. **a** Total RNA was extracted from normal nasopharyngeal epithelium cells (NP69) and NPC cell lines (HNE1, HNE2, CNE2, C666-1, HK-1) and reversely transcribed into cDNA. Q-PCR was performed and analyzed for the expression level of miR-29c normalized by RNAU6. **b** The same method and protocol was performed from NPC tissue and reversely transcribed, Q-PCR was performed and analyzed. N: normal nasopharyngeal epithelium tissue, 2, 3, 4 were presented for Clinical Stage 2, 3, 4, M was presented for the NPC tissue with metastasis. 30 samples were used, each group contains six samples

To investigate our hypothesis, we first examined the expression of miR-29c in NP69, HNE-1, HNE2, CNE2, HK1, and C666-1 cells. As previously reported, miR-29c is relative high in normal nasopharyngeal epithelial cells (NP69), and low in NPC cell lines (HNE-1, CNE2, HK1, HNE2, C666-1) (Fig. 1a). In the tissues of NPC patients, snap-frozen NPC biopsies were obtained from NPC patients and normal healthy nasopharyngeal epithelial samples from biopsy-negative cases were used as control. The criteria of clinical staging of NPC samples were based on the 2008 staging system of NPC and AJCC staging system [16, 17]. Samples were first frozen-sectioned by using a LEICA CM 1900 cryomicrotome. 6 NPC samples in each clinical staging II ~ V were used (numbers I to IV) and control group. Samples were collected from the Second Xiangya Hospital affiliated by Central South University. The patients were informed about the sample collection and had signed informed consent forms. Collections and use of tissue samples were approved by the ethical review committees of Xiangya Second Hospital. Laser capture micro-dissection was used to separate the cancer tissues from the normal tissues [18]. Phase contrast images were acquired using LEICA CTR 6500 microscope. Total RNA was extracted using Trizol® reagent (Invitrogen) from samples. Two hundred nanograms (200 ng) of total RNA from each sample were used for the follow-up microarray. As the result showed the expression level of miR-29c is negatively associated with clinical stage (Fig. 1b).

### MiR-29c increases the expression level of miR-34b/c and miR-449a significantly

**Fig. 2:**
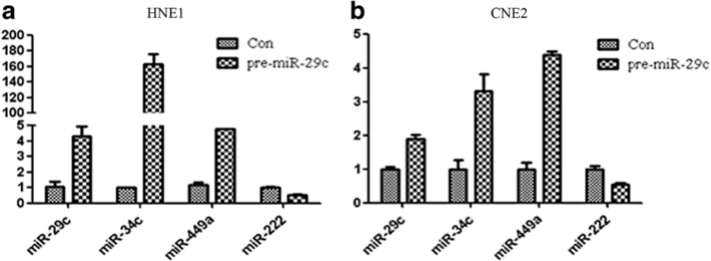
MiR-29c increases the level of miR-34c and miR-449a but not miR-222 in nasopharyngeal carcinoma cell lines. The pSuper-pre-miR-29c was transformed into nasopharyngeal carcinoma cells HNE1 (**a**) and CNE2 (**b**) according to the protocol and the cells were cultured for 24 h. Total RNA was extracted and inversely transcribed into cDNA. Q-PCR was performed and analyzed for the miR-29c, miR-34c, miR-449a and miR-222 normalized by RNAU6

**Fig. 3:**
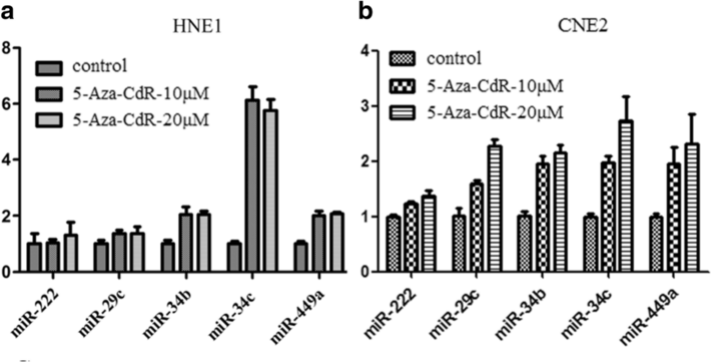
miR-34b/c and miR-449a levels were regulated by the epigenetic factors but miR-222 wasn’t. Nasopharyngeal carcinoma cells were treated with 5-Aza-CdR for 72 h, and then with or without Trichostatin A (TSA) for another 24 h. **a**, **b** Expression of miR-34b/c and miR-449a were analyzed

Pre-miR-29c cDNA or scramble DNA was inserted into pSuper vector and confirmed to be correct by sequencing. The constructs were transfected into nasopharyngeal carcinoma cell line HNE1 and CNE2 in which miR-29c expression is down-regulated. It is surprisingly found that the expression of miR-34c and miR-449a were increased, while that of miR-222 wasn’t altered (Fig. 2a and b). In mammalian genome, the miR-34 family (miR-34 s) consists of miR-34a, miR-34b and miR-34c. miR-34a localizes to chromosome 1p36, while miR-34b and miR-34c form a cluster and localize to chromosome 11q23. In additional experiments, miR-29c mimics and negative control reagents(Qiagen, German) were transfected into the cell lines, we got similar results (data not shown). Mir-34c and miR-449a belong to miR-34 family which is found down-regulated in nasopharyngeal carcinoma and other cancers [18–21]. Expression of miR-34 family members were reported down-regulated in cancer cells by abnormal DNA methylation [21–28]. However, the molecular mechanism of miR-34c/miR-449a down-regulation in nasopharyngeal carcinoma is not clear. In order to explore whether the expression of miR-34c and 449a in nasopharyngeal carcinoma cells is regulated by epigenetic factors, the cells were treated with DNA methylation inhibitor, 5-Aza-2′-deoxycytidine (5-Aza-CdR), or/and histone deacetylase inhibitor, Trichostatin A (TSA). As expected, the expression of miR-34b/c and 449a is increased with the treatment of 5-Aza-CdR and that of miR-222 was not altered either in HNE1 and CNE2 (Fig. 3a and b). To update, there has no literatures indicating that miR-222 is regulated by epigenetic factors, which may explain the reason why miR-222 expression wasn’t affected by 5-Aza-CdR. Several software analysis also showed no typical CpG islands exists in the genomics sequences of miR-222 or miR-29c. DNA methyltransferase 3a and 3b expression were inhibited by 5-Aza-CdR treatment, while DNA methyltransferase T1 seemed not to be altered (Fig. 3c and d).

### DNMT3a and 3b are direct targets of miR-29c

**Fig. 4:**
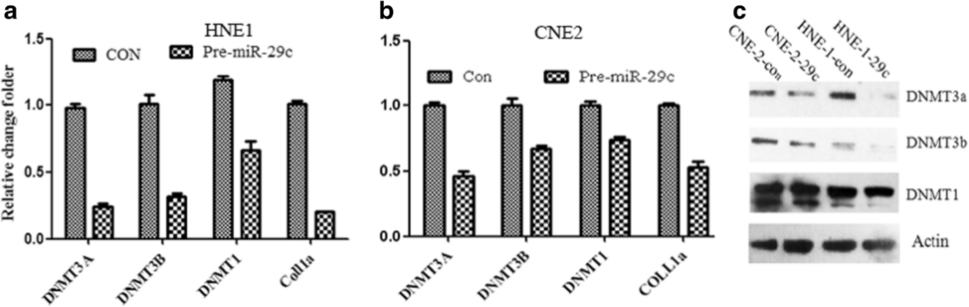
DNMT3a and 3b but not DNMT1 are down-regulated by pSuper-pre-miR-29c transfection. DNMT3a, 3b, 1 were predicted as tentative targeted genes of miR-29c. Cells were transformed with pre-miR-29c and cultured for 24 h for Q-PCR and western blots for 48 h. **a**, **b** Q-PCR analysis of DNMT3a, 3b, 1 regulated by miR-29c in HNE1 and CNE2. **c** Western blot analysis of DNMT3a, 3b and DNMT1 in different cell lines transfected with pre-miR-29c

In order to determine whether miR-29c regulate the miR-34c and 449a through down-regulating the DNMT3a and DNMT3a, pre-miR-29c constructs or hsa-29c mimics and scramble DNA were transfected into HNE1 and CNE2 cell line, respectively. It was found that the expression of DNMT3a and DNMT3b were decreased significantly with the transfection pre-miR-29c or hsa-29c mimics, but not altered with scramble constructs. However, the level of DNMT1 was not altered significantly (Fig. 4a, b and c). The miR-29 family members have intriguing complementarities to the 3′-UTRs of DNMT-3a and -3b, involved in DNA methylation. DNMT3a and 3b have been confirmed as direct targets of miR-29c in lung carcinoma, breast cancer, and cutaneous melanoma [29–32]. The expression of miR-29 family members are inversely correlated to DNMT-3a and -3b in lung cancer, directly targeting both DNMT3a and -3b [32–33].

### Expression of DNMT3a, 3b, T1 associated with prognosis of nasopharyngeal carcinoma

**Fig. 5:**
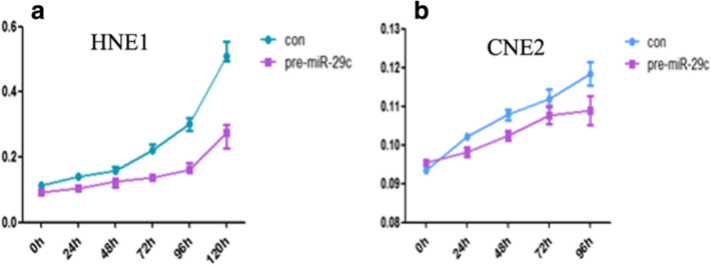
MiR-29c inhibits the growth of nasopharyngeal carcinoma cells HNE1 and CNE2 by MTT assay. Cells were transformed with pre-miR-29c and cultured for 24 h. 5 × 10^4^ cells were seeded into 96-well dish triplicately. 20ul MTT solution was added to each well and then 200ul DMSO was added to the well with cells. Read optical density at 490 nm and subtract background at 570 nm. The readout was recorded at 4 time points (24, 48, 72, 96,120 h)

**Fig. 6:**
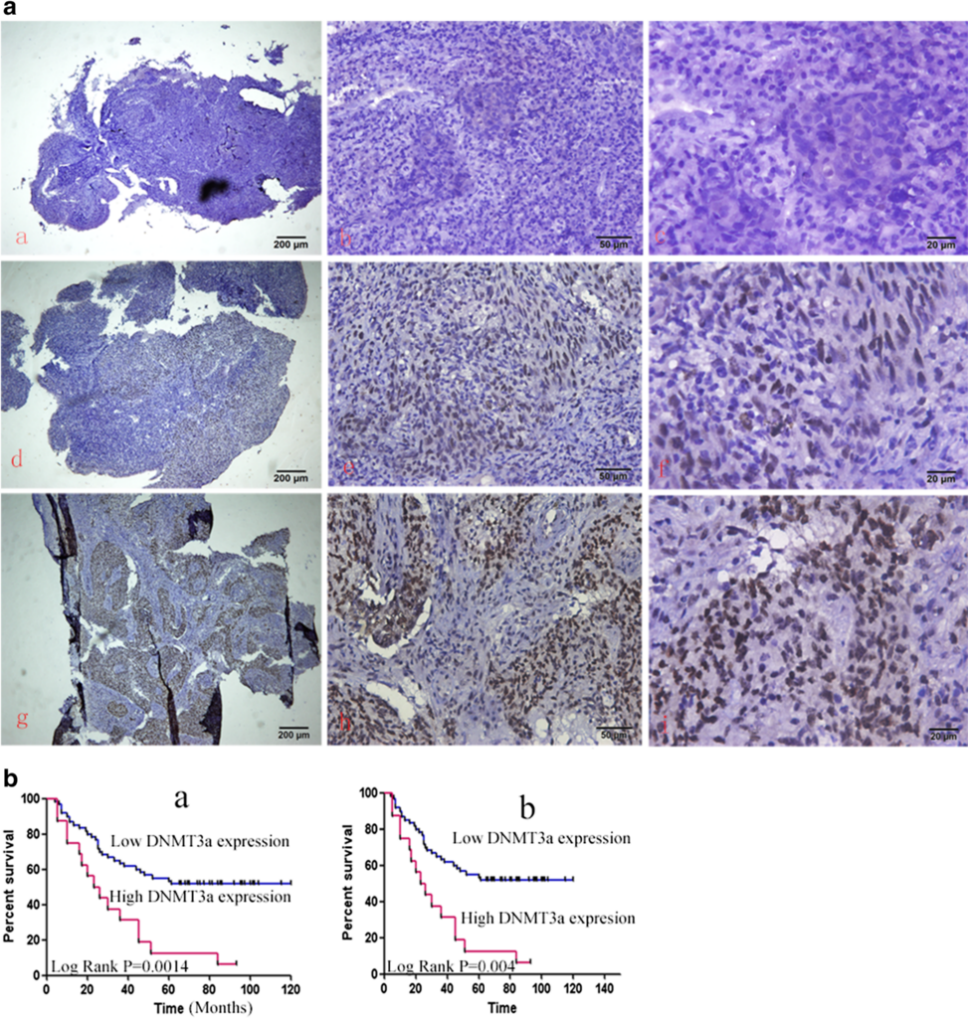
Representative image of IHC staining of DNMT3a in nasopharyngeal carcinoma tissue. **a** Negative (−) (*a-c*), weak (+) (*d-f*), positive (++) (*g-i*) staining of DNMT3a in NPC tissue. **b** The correlation of DNMT3a staining with 5-year survival (*a*) and total survival time (*b*). * *p* < 0.05 is considered to be significant statistically

**Fig. 7:**
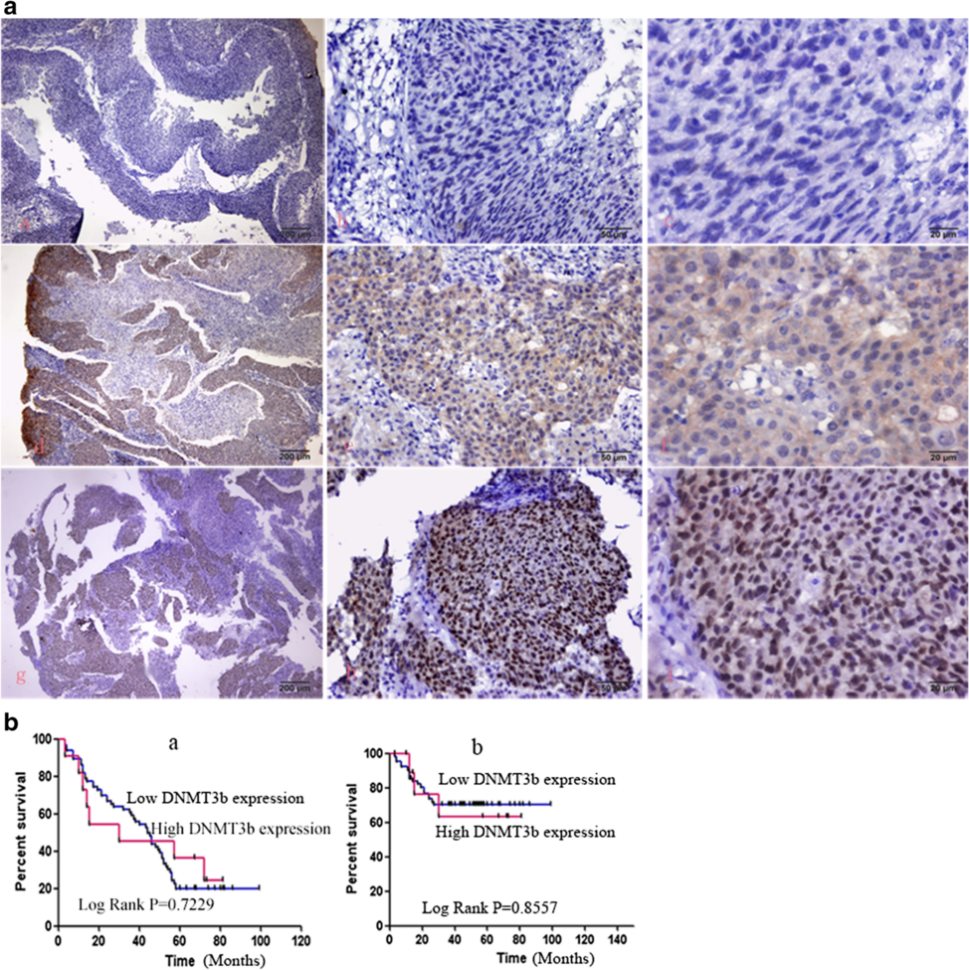
Representative image of IHC staining of DNMT3b in nasopharyngeal carcinoma tissue. **a** Negative (−) (*a–c*), weak (+) (*d–f*), positive (++) (*g–i*) staining of DNMT3b in NPC tissue. **b** The correlation of DNMT3b staining with 5-year survival (*a*) and total survival time (*b*). * *p* < 0.05 is considered to be significant statistically

**Fig. 8:**
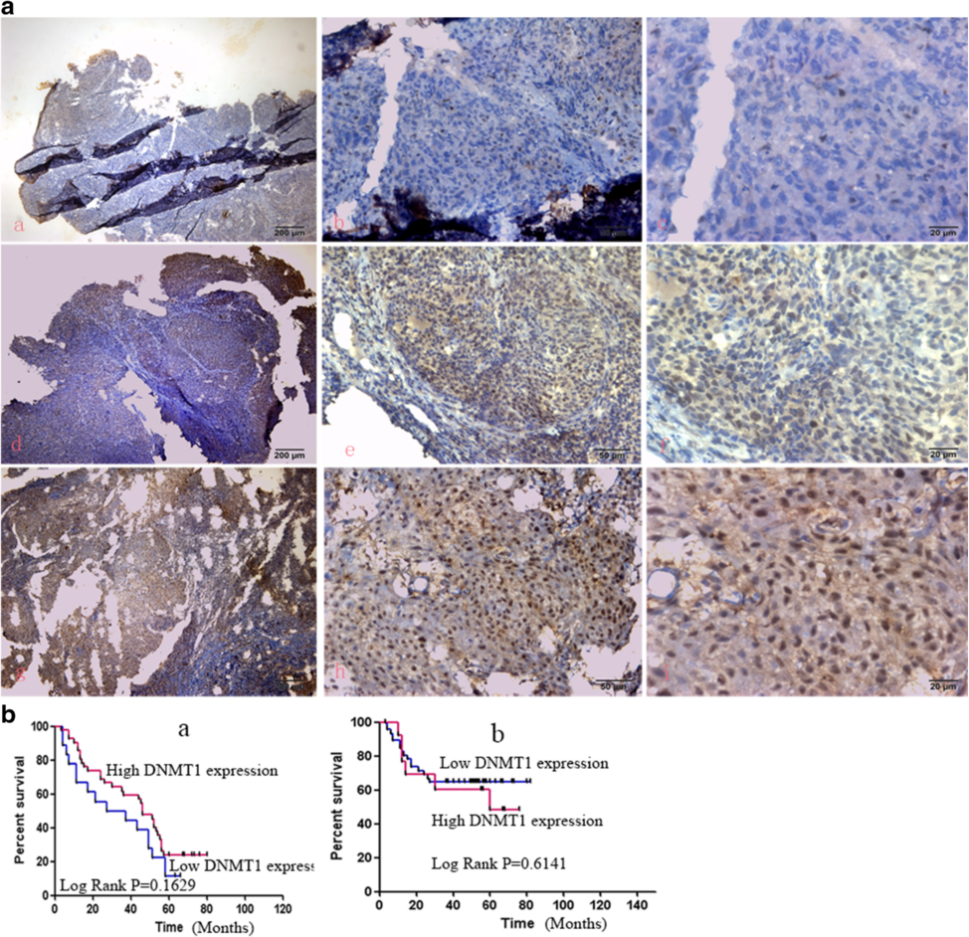
Representative image of IHC staining of DNMT1 in nasopharyngeal carcinoma tissue. **a** Negative (−) (*a–c*), weak (+) (*d–f*), positive (++) (*g–i*) staining of DNMT1 in NPC tissue. **b** The correlation of DNMT1 staining with 5-year survival (*a*) and total survival time (*b*). * *p* < 0.05 is considered to be significant statistically

Based on microarray analysis in previous study it has been found that miR-29c,miR-34c, and miR-449a are down-regulated in NPC (data not shown). The target genes of miR-29c such as BCL2L2, HBEGF, HBP1, HSPG2, ITGB1, LAMC2, LTBR, MIB1, MLF1, MMP2,NDST1,SVEP1MCL-1,BCL-2,TIAM1 were up-regulated and miR-29c could sensitize NPC cells to ionizing radiation and cisplatin treatment by promoting apoptosis [10, 11, 18]. In our study, the recovery of miR-29c expression delayed the proliferation and growth of NPCs (Fig. 5a and b). We examined that DNMT3a, 3b and T1 are strongly expressed in NPC tissues. The clinical information of the patients was listed in Tables 1, 2 and 3. DNMT3a, 3b, 1 expression is not associated with gender (*p* = 0.0652, 0.2127, 0.7638 respectively) or age (*p* = 0.0557, 0.5747, 0.7679, respectively). The expression of DNMT3a, but neither DNMT3b nor DNMT1 was associated with clinical stage of NPC (*p* = 0.0012, *p* = 0.3122 and 0.6202 respectively). The representative images of positive and negative expression of DNMT3a, 3b and T1 are shown in Fig. 6a (a–i), 7A (a–i), 8A (a–i). The score was evaluated by 3 experienced pathologists were analyzed with *χ*2 test. The expression of DNMT3a is negatively associated with 5-year survival time (log Rank *p* = 0.0014) and total survival time (Fig. 6B a–b), however, the expression of DNMT3b, DNMT1 are not significantly associated with 5-year survival time and total survival time (Fig. 7ba–b, Fig. 8ba–b).

**Table 1:** Correlation between DNMT3a expression and clinicopathological characteristics of nasopharyngeal carcinoma

Variable	No. of patients (n, %)	DNMT3a		Chi-squared test
		Low Exp (n, %)	High Exp (n, %)	*P*-value
Age (years)				
<45	25	22 (88)	3 (12)	0.0557
>45	43	32 (74.4)	11 (25.6)	
Gender				
Male	54	43 (79.6)	11 (20.4)	0.0652
Female	14	10 (71.4)	4 (28.6)	
Stage				
TNM I-II	16	13 (81.2)	3 (18.8)	0.0012^*^
TNM III-IV	39	27 (69.2)	12 (30.8)	
Unknown	13	10 (76.9)	3 (32.1)	

*p < 0.05 was significant statistically

**Table 2:** Correlation between DNMT3b expression and clinicopathological characteristics of nasopharyngeal carcinoma

Variable	No. of patients (n, %)	DNMT3b		Chi-squared test
		Low Exp (n, %)	High Exp (n, %)	*P*-value
Age (years)				
<45	40	33 (82.5)	7 (17.5)	0.5747
>45	38	32 (84.2)	6 (15.8)	
Gender				
Male	64	54 (84.4)	10 (15.6)	0.2127
Female	14	14 (100)	0	
Stage				
TNM I-	19	13 (68.4)	6 (31.6)	0.3122
TNM III-IV	47	40 (85.1)	7 (14.9)	
Unknown	12	11 (91.7)	1 (8.3)

**Table 3:** Correlation between DNMT1 expression and clinicopathological characteristics of nasopharyngeal carcinoma

Variable	No. of patients (n, %)	DNMT1		Chi-squared test
		Low Exp (n, %)	High Exp (n, %)	*P*-value
Age (years)				
<45	29	21 (72.4)	8 (27.6)	0.7679
>45	31	24 (77.4)	7 (22.6)	
Gender				
Male	47	34 (72.3)	13 (27.7)	0.7638
Female	13	9 (69.2)	4 (30.8)	
Stage				
TNM I-II	12	9 (75)	3 (25)	0.6202
TNM III-IV	38	25 (65.8)	13 (34.2)	
Unknown	10	9 (90)	1 (10)

## Discussions

As well known, miRNAs play an important role in various cellular activities by regulating gene expression of their targets. Recent studies have shown that the expression of miRNA is regulated by epigenetic modifications by DNA methylation or histone modification. MiRNA also can be the key factor to regulate the levels of DNA methylation or histone modification which affect the expression level of other molecules. All these factors (extracellular signals, miRNAs, transcription factor, targeted gene) are the members of the vast gene expression regulatory networks. In cancer cells, the epigenetic modifications of miRNAs have been reported. Those miRNAs acting as tumor suppressor often were silenced by frequent hypermethylation or histone deacetylation. Furthermore, it shows tumor specialty. When treated with demethylating agent 5-aza-deoxycytidine (5-Aza-CdR) and histone deacetylase (4-Phenylbutyrie acid, PBA), the expression of 5 % miRNAs in bladder carcinoma cell line T24 increased by 3 folder than that of untreated. MiR-34c acts as a suppressor in many tumors. It’s down-regulated and the target genes DCBLD2, FOXN3, IKZF1, NPTN PAFAH1B1, USP10, YY1, ARHGAP1, ARHGEF3, BCL11B, C16orf5, CNTNAP1, FOXN3, FUT8, IL6R, ITGB8, ITSN1, JAG1, MLL2, NDST1,NOTCH2, NPNT, PPFIA1, PTPRM, PVRL1, SERPINE1, VCL were up-regulated in NPC [18]. Single hyper methylation of CpG island in the promoter region of miR-34c gene repressed miR-34c expression by reducing DNA binding activities of Sp1 and promoted self-renewal and epithelial-mesenchymal transition of breast tumor-initiating cells [32]. Differential methylation of CpG islands neighboring the miR-34c promoter inhibited the expression of miR-34c in gastric cancer cell lines and in paclitaxel-resistant gastric cancer samples. MiR-34c was down-regulated and its target microtubule-associated protein tau (MAPT) protein expression was high. Over expression of miR-34c significantly down-regulated MAPT protein expression and increased the chemo sensitivity of paclitaxel-resistant gastric cancer cells [34]. Aberrant DNA methylation of miR-34c was correlated with a high probability of recurrence and associated with poor overall survival and disease-free survival in non-small cell lung cancer [35, 36]. MiR-449a was also found to be down-regulated in NPC [20]. MiR-449a can directly target HDAC1 in primary lung cancer and inhibit cell growth and anchorage-independent growth [37]. Trichostatin A (TSA) could strongly increase miR-449a levels in testicular cancer cell lines and miR-449a down-regulated the histone deacetylase Sirt1 [38]. These studies manifest that miR-34c and miR-449a were regulated by the epigenetic factors. According to our previous data, miR-29c, miR-34c, miR-449a were down-regulated in NPC. In this study, we treated the NPC cell line HNE-1 and CNE-2 by 5-AzadC for 96 h and then found that miR-34c and miR-449a increased. MiR-34c and miR-449a were associated with cell proliferation, apoptosis, anti-tumor drug resistance and serum biomarkers of recurrence in other cancers, this new miRNA-miRNA pathway may provide a new sight on the diagnosis, treatment and prognosis of NPC.

## Conclusions

In a summary, we found that miR-29c was further confirmed to be down-regulated in NPC cell lines HNE-1, CNE2, C666-1 and tissues, and firstly reported that restoration of miR-29c increases the expression miR-34c and miR-449a which were regulated by DNA methytranferases through epigenetic factors. Our experiments verified that DNMT-3a and -3b are the targets of miR-29c. As epigenetic regulation is reversible, the effects can be available through specific drugs such as DNMT inhibitors (5-Aza-CdR) with or without HDAC inhibitor (TSA). Another kind of strategy, oligonucleotides (synthetic miRNA oligonucleotides) can be used directly in vivo to correct the disorders in miRNA expression levels, which is expected as a new therapeutic tool for the nasopharyngeal carcinoma patients.
